# Subcutaneously administered Menopur(R), a new highly purified human menopausal gonadotropin, causes significantly fewer injection site reactions than Repronex(R) in subjects undergoing in vitro fertilization

**DOI:** 10.1186/1477-7827-3-62

**Published:** 2005-11-09

**Authors:** William R Keye, Bobby Webster, Richard Dickey, Stephen Somkuti, Jack Crain, M Joseph Scobey

**Affiliations:** 1 William Beaumont Hospital, In Vitro Fertility Clinic, Royal Oak, Michigan, USA; 2 Woman's Center for Fertility, Baton Rouge, Louisiana, USA; 3 Fertility Institute of New Orleans, New Orleans, Louisiana, USA; 4 Abington Reproductive Medicine, Abington, Pennsylvania, USA; 5 Reproductive Endocrine Associates of Charlotte, Charlotte, North Carolina, USA; 6 Ferring Pharmaceuticals Inc., Suffern, New York, USA

## Abstract

**Background:**

The safety and tolerability of a new highly purified, urine-derived human menopausal gonadotropin (hMG) preparation [Menopur(R)] was compared with a currently available hMG [Repronex (R)] in women undergoing in vitro fertilization (IVF).

**Methods:**

This was a randomized, open-label, parallel-group, multicenter study conducted in subjects undergoing IVF. Women (N = 125), 18–39 years of age, underwent pituitary down-regulation with leuprolide acetate beginning 7 days prior to onset of menses and continuing up to the day before hCG administration. Subjects were randomized to receive subcutaneous (SC) Menopur (R) (n = 61) or Repronex (R) SC (n = 64) for a maximum of 12 days. All adverse events (AEs) were recorded and subject self-assessments of injection site reactions were recorded in a daily diary.

**Results:**

Significantly fewer subjects in the Menopur (R) group reported injection site reactions (P < 0.001) compared to the Repronex (R) group. Overall, there was no statistically significant difference in the incidence of AEs between the two treatment groups.

**Conclusion:**

Menopur (R) SC offers a greater safety and tolerability profile compared to Repronex (R) SC.

## Background

Zondek and colleagues were the first to propose that the pituitary gland secretes hormones that stimulate the gonads [1]. This hypothesis was later confirmed with the identification of two different hormones, follicle-stimulating hormone (FSH) and luteinizing hormone (LH) [2]. These advances in the understanding of the human reproductive process converged in 1958 with the successful clinical use of pituitary gonadotropins to induce ovulation in anovulatory women [3].

Development of simple extraction and purification techniques led to the production of human menopausal gonadotropins (hMG) in quantities sufficient for clinical use. In 1962, the first pregnancy resulting from the use of a urine-derived hMG for follicular stimulation was reported [4]. Since then, human-derived gonadotropins have remained a reliable and safe treatment for infertility. However, the purity of early hMG preparations was low and the majority of the injectant preparations consisted of uncharacterized urinary proteins [5]. The uncharacterized proteins produced adverse injection site reactions when administered intramuscularly (IM) and the product could not be administered subcutaneously (SC). Newer purification techniques applied to the manufacturing of hMG resulted in enhanced purity and enabled SC administration. However, mild to moderate injection site reactions were still common. Most recently, modern day purification techniques have resulted in the availability of a new, high purity hMG preparation that has nearly eliminated injection site reactions.

The purification process for Repronex^® ^included at least 24 steps involving adsorptions, dialysis and precipitations, as well as ionic, cationic, and hydrophobic exchange chromatography [6]. Advances in these manufacturing techniques and the inclusion of additional purification steps have now produced a new highly purified hMG (Menopur^® ^, 75 IU LH:75 IU FSH; Ferring Pharmaceuticals Inc., Suffern, New York) that is nearly devoid of uncharacterized proteins. This high purity hMG was developed to reduce the frequency of injection site reactions. Therefore, we conducted a clinical trial to compare the safety and tolerability of Menopur^® ^with the currently available hMG preparation, Repronex^® ^(75 IU LH:75 IU FSH; Ferring Pharmaceuticals Inc.) in women undergoing controlled ovarian hyperstimulation (COH) for in vitro fertilization (IVF).

## Methods

This was a randomized, open-label, parallel-group, multicenter study comparing one cycle of treatment with Menopur^® ^SC or Repronex^® ^SC at a dose of 75 to 450 IU/day administered as a single, daily injection for up to 12 days in infertile women undergoing COH for IVF. Each subject participated in only one cycle of IVF. Fifteen centers participated in the study, each of which obtained institutional review board approval. Written informed consent was obtained from all participants prior to screening and study enrollment.

### Subjects

Subjects had to meet the following eligibility criteria to participate in the study: nonsmoking, 18 to 39 years of age with regular ovulatory cycles (24 to 35 days); have a diagnosis of unexplained infertility or infertility due to tubal factor; stage I or II endometriosis; normal ovaries and uterus on transvaginal ultrasonography; normal serum levels of estradiol (E_2_), prolactin, LH, FSH, testosterone, dehydroepiandrosterone sulfate, and thyroid-stimulating hormone; and have a body mass index of ≤ 34. In addition, all subjects were seronegative for hepatitis B and C and HIV and had a negative pregnancy test prior to initiating treatment. A semen analysis performed on a sample from either the subject's partner or the designated donor had to be normal according to the criteria established by the World Health Organization. A minimum of one menstrual cycle without IVF/assisted reproductive therapy (ART) treatment was required prior to screening. Subjects were excluded from participation if there was evidence of any clinically relevant systemic disease or any surgical or medical condition that could interfere with the absorption, metabolism, or excretion of gonadotropins. Subjects were not to have had a positive pregnancy test within three months of baseline screening and were also excluded from the study if they had undergone three or more prior ART cycles, had abnormal uterine bleeding, a history of substance abuse, a history of chemotherapy, were breast feeding, or if they had participated in any experimental drug study within 60 days of screening for this study.

### Protocol

Each eligible subject received daily injections of leuprolide acetate (LA; TAP Pharmaceuticals, Deerfield, IL; 0.5 mg/d SC) beginning 7 days prior to the anticipated onset of menses until the day before administration of human chorionic gonadotropin (hCG)(Novarel™, Ferring Pharmaceuticals Inc., Suffern, NY). LA was continued until there was evidence of down-regulation as indicated by a serum E_2 _concentration of ≤ 45 pg/mL and an endometrial lining ≤ 7 mm on transvaginal ultrasound. If the E_2 _level was ≥ 45 pg/ml, the endometrial lining was > 7 mm, or menses did not occur within 20 days after beginning LA, the subject was withdrawn from the study.

Subjects who met the down-regulation criteria listed above were randomized to receive single, daily doses of Menopur^® ^SC or Repronex^® ^SC. They were instructed to self-administer hMG SC, alternating their injections between the right and left lower abdomen at approximately the same time every afternoon. Subjects were also instructed to record injection site pain each day using a 0- to 10-point scale, with 0 representing no pain and 10 representing extreme pain. In addition, subjects recorded all adverse events (AEs) experienced during the study, including those associated with injection site reactions. Subjects received 225 IU for 4 days and on day 5, subjects returned to the study centers for transvaginal ultrasound and determination of E_2 _levels. Based on these findings, the investigators adjusted the daily dose of hMG by 75 to 150 IU up to a maximum of 450 IU per day. Serum E_2 _levels and ultrasound measurements were taken prior to each dose escalation and a maximum of 12 days total hMG treatment was allowed. Investigators were permitted to decrease the hMG dose at any time based on clinical judgment and safety concerns, and could discontinue hMG and/or withhold hCG administration if they believed the subject was at risk for development of ovarian hyperstimulation syndrome (OHSS). When at least 3 follicles reached a diameter of ≥ 16 mm as measured by transvaginal ultrasound and E_2 _levels were appropriate for the number of follicles observed based on the investigator's clinical judgment, hMG was discontinued and hCG (Novarel™, Ferring Pharmaceuticals, Inc.) was administered IM at a dose of 10,000 USP units. Oocytes were retrieved 34 to 36 hours later. Standard center-specific IVF culture conditions were allowed, however intracytoplasmic sperm injection (ICSI) and assisted hatching were not. Study centers were permitted to use coculture with homologous cells if it was standard practice and routinely used in all subjects undergoing IVF at that center. A maximum of four embryos could be transferred. Progesterone (Crinone™ 8% gel, 90 mg qd, Serono Laboratories, Inc., Randolph, MA) was self-administered beginning on day 2 or day 3 after oocyte retrieval for luteal phase support, and continued until there was fetal heart motion in an intrauterine pregnancy or there was a negative serum pregnancy test (β-hCG).

Throughout the study, investigators recorded the presence and nature of any AEs. Within 3 weeks of the initial β-hCG quantitative serum pregnancy test or discontinuation from study, subjects returned to the study center for an exit physical examination. Subject reports of AEs were recorded on the case report form and tabulated using Coding Symbols for Thesaurus of Adverse Reaction Terms (COSTART) terminology. Subjects' daily diary evaluations of injection site pain were tabulated on a 0- to 10-point scale.

### Statistical evaluation

The primary efficacy of this study was oocytes retrieved and therefore power calculations were done with 80% power to detect an among group difference of 30% in the number of oocytes retrieved. This required 59 patients per group.

A chi-square test was used to make between group comparisons on the percentage of subjects with at least one AE and at least one mild to moderate AE while a Fisher exact test was used to make comparisons on the percentage of subjects with at least one severe AE and at least one serious AE. A chi-square test was used to test for differences in the percentage of subjects with abnormal findings recorded at the study exit physical examination, while the Fisher exact test was used to test for differences in the percentage of subjects with clinically significant abnormal findings. The initial analysis of injection site pain on each treatment day compared the two treatment groups using a one-way ANOVA. A linear mixed model was then used to make treatment comparisons of injection site pain throughout the study. This model allowed the analysis of continuous correlated data to account for within subject variation.

## Results

A total of 190 subjects were randomized and included in the analysis of safety. The initial study contained a third arm consisting of 65 subjects who received Menopur^® ^IM, however these data were not included in this analysis, as the focus of this report is to compare the safety and tolerability of Repronex^® ^SC and Menopur^® ^SC. The remaining 125 subjects were randomized to receive Menopur^® ^SC (n = 61) or Repronex^® ^SC (n = 64). Due to subject noncompliance or loss at follow-up, certain safety outcomes such as exit physical examination variables and injection site pain are missing a few data points.

Subject demographic characteristics are summarized in Table 1. Overall, subjects in the two treatment groups were comparable both demographically and medically. The only statistically significant difference between the groups was race, with African-Americans comprising 11.5% of the Menopur^® ^group compared with 1.6% of the Repronex^® ^group (P = 0.039). The impact of this difference is unknown.

**Table 1:** Demographic Characteristics of Subjects*

**Characteristic**	**Menopur^® ^(n = 61)**		**Repronex^® ^(n = 64)**	
Age (yrs)	32.3	(3.7)	32.5	(4.1)
Weight (kg)	63.5	(11.0)	63.5	(10.0)
Height (cm)	161.5	(7.4)	163.3	(6.4)
Body mass index (kg/m^2 ^)	24.4	(3.6)	24.0	(3.4)
Race, no. (%)				
Caucasian	47	(77.0)	54	(84.4)
African-American	7	(11.5)	1	(1.6)
Asian	0	(0)	2	(3.1)
Hispanic	5	(8.2)	5	(7.8)
Native American	0	(0)	0	(0)
Other	2	(3.3)	2	(3.1)

*Values are mean (SD).

There were no statistically significant differences between the treatment groups in the number of subjects with any AEs, severe AEs, or serious AEs, as shown in Table 2. There were five serious AEs during the study (1 subject in the Menopur^® ^group had OHSS and four subjects in the Repronex^® ^group had one of the following serious AEs: dehydration, an ectopic pregnancy, a right ruptured ovary with secondary hemothorax, and a pelvic abscess). A total of three cases of OHSS were reported (1 subject in the Menopur^® ^group, which was severe and 2 subjects in the Repronex^® ^group, which were mild or moderate).

**Table 2:** Subjects with Adverse Events*

**Adverse Event**	**Menopur^® ^(n = 61)**		**Repronex^® ^(n = 64)**		**P Value**
Any	41	(67.2)	48	(75.0)	0.620
Severe	5	(8.2)	5	(7.8)	0.402
Serious	1	(1.6)	4	(6.3)	0.456

*Values represent numbers (percentage) of subjects with one or more adverse event.

**Figure 1:**
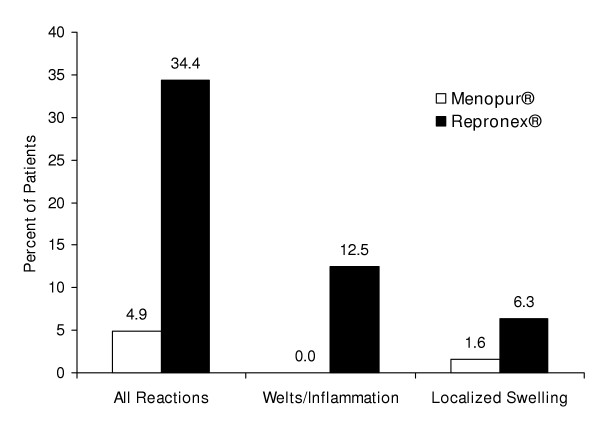
**Subjects with hMG-Associated Injection Site Reactions**. This figure shows the percentage of subjects with any hMG associated injection site reaction as well as those with reactions that included welts or inflammation and those whose reactions involved swelling.

Table 3 lists the AEs with an incidence of ≥ 5% (2 or more subjects). Among these AEs, there were no significant differences between the two groups in the percentage of subjects with any AE and no difference in the intensity of injection site pain. However, there were numerically fewer total AEs in the Menopur^® ^group (n = 131) compared to the Repronex^® ^group (n = 198). As shown in Figure 1, this difference was largely attributed to the number of injection site reactions, the single most common AE. When only hMG injections were considered, there were only three (4.9%) subjects in the Menopur^® ^group that reported injection site reactions, whereas 22 (34.4%) subjects in the Repronex^® ^group reported injection site reactions (P < 0.001). Among the three Menopur^® ^subjects with local injection site reactions, all were transient and mild to moderate in intensity, none developed welts/inflammation, and only one subject had localized swelling. These findings contrasted with the 22 subjects in the Repronex^® ^group with injection site reactions, among whom eight developed welts/inflammation (P < 0.001) and four developed swelling (P = 0.328). Conversely, there was no difference in mean scores for injection site pain between the two groups, 2.6 for Menopur^® ^and 2.3 for Repronex^® ^(P = 0.615).

**Table 3:** Subjects with Adverse Events: Incidence Rate ≥ 5%* (two or more subjects)

**Adverse Event**	**Menopur^® ^n = 61**		**Repronex^® ^n = 64**	
Abdominal cramps	13	(21.3)	14	(21.9)
Headache	13	(21.3)	13	(20.3)
Post retrieval pain	7	(11.5)	9	(14.1)
Nausea	6	(9.8)	10	(15.6)
Vaginal spotting	6	(9.8)	5	(7.8)
Abdominal fullness	5	(8.2)	7	(10.9)
Abdominal pain	5	(8.2)	4	(6.3)
Constipation	5	(8.2)	1	(1.6)
Respiratory disorder	4	(6.6)	4	(6.3)
Vaginal hemorrhage	2	(3.3)	4	(6.3)
Breast tenderness/pain	2	(3.3)	5	(7.8)
Malaise	2	(3.3)	4	(6.3)
Sinusitis	2	(3.3)	4	(6.3)

*Values represent numbers (percentage) of subjects with adverse event.

## Discussion

Overall, the safety profile of Menopur^® ^in this study was similar to that of Repronex^® ^. Human-derived gonadotropins have been used safely and effectively in ART protocols for over forty years. However, the injection of partially purified hMG is associated with more injection site reactions than highly purified gonadotropins. Removal of nearly all uncharacterized proteins from hMG in the manufacturing process for Menopur^® ^has resulted in significantly fewer reported injection site reactions in IVF subjects. There was a seven-fold difference in the percentage of subjects with injection site reactions, 4.9% and 34.4% of subjects in the Menopur^® ^and Repronex^® ^groups, respectively. When the incidence of reactions that involved swelling, inflammation, or welts was examined, 98% of subjects receiving Menopur^® ^completed their cycle without such reactions while only 81% of subjects receiving Repronex^® ^did not experience such events (P = 0.001).

An analysis of Menopur^® ^has shown that its purity and quality is comparable to recombinant gonadotropin preparations [7]. In addition, Menopur^® ^has been shown to have a similar safety and tolerability profile as recombinant FSH in women undergoing IVF/ICSI treatment cycles [8]. Collectively, these observations and studies, combined with the data from this study demonstrate that Menopur^® ^is at least as efficacious and safe as any existing gonadotropin.

The results from this study demonstrate that Menopur^® ^, a new highly purified hMG, can be administered SC with significantly fewer injection site reactions than Repronex^® ^, a partially purified hMG. Thus, advanced manufacturing techniques have produced the first ever highly purified form of hMG resulting in a markedly improved safety and tolerability profile compared with previously available hMG products.

## Authors' contributions

Drs. Keye, Webster, Dickey, Somkuti, and Crain contributed to the treatment of subjects, collection of data and writing of the manuscript. Dr. Scobey was instrumental in data analysis and writing of the manuscript.
